# Supplementary material for: Interfacial Interaction Enhanced Rheological Behavior in PAM/CTAC/Salt Aqueous Solution—A Coarse-Grained Molecular Dynamics Study
Source: Polymers (Basel). 2020 Jan 25;12(2):265. doi: 10.3390/polym12020265 (PMC7077399; doi:10.3390/polym12020265)

polymers

Supporting Information

Interfacial Interaction Enhanced Rheological Behavior in PAM/CTAC/Salt Aqueous Solution- A Coarse-Grained Molecular Dynamics Study

Dongjie Liu[1,3], Yong Li [2], Fei Liu [3], Wenjing Zhou [1], Ansu Sun [4], Xiaoteng Liu [4], Fei Chen [1,*], Ben Bin Xu [4,*] and Jinjia Wei[1,3,*]

1 School of Chemical Engineering and Technology, Xi'an Jiaotong University, Xi'an 710049, China; jun080511@stu.xjtu.edu.cn (D.L.); wj.zhou@mail.xjtu.edu.cn (W.Z.)
2 Drilling and Production Engineering Research Institute, Chuanqing Drilling and Exploration Engineering Company Ltd, CNPC, Xi'an 710018, China; liyong_gcy@cnpc.com.cn (Y.L.)
3 State Key Laboratory of Multiphase Flow in Power Engineering, Xi'an Jiaotong University, Xi'an 710049, China; bestceo@xjtu.edu.cn (F.L.)
4 Mechanical and Construction Engineering, Faculty of Engineering and Environment, Northumbria University, Newcastle upon Tyne, NE1 8ST, UK; terence.liu@northumbria.ac.uk (T.L.); ben.xu@northumbria.ac.uk (B.B.X.);
* Correspondence: feichen@xjtu.edu.cn, (F.C.); ben.xu@northumbria.ac.uk (B.B.X.); jjwei@xjtu.edu.cn (J.W.); Tel.: +86-029-82664375 (J.W.)

Table S1. Nonbonded interaction parameters.

	Q_a	Q_0	C_2	C_1	SC_1	SC_4	Q_d	P_4	P5	BP_4
Q_a	5.0	4.5	2.0	2.0	2.0	2.7	5.6	5.6	5.6	5.6
Q_0	0.47	3.5	2.0	2.0	2.0	2.7	4.5	5.6	5.0	5.6
C_2	0.62	0.62	3.5	3.5	3.5	3.1	2.0	2.3	2.3	2.3
C_1	0.62	0.62	0.47	3.5	3.5	3.1	2.0	2.0	2.0	2.0
SC_1	0.62	0.62	0.47	0.47	2.625	2.325	2.0	2.0	2.0	2.0
SC_4	0.43	0.47	0.47	0.47	0.43	2.6	2.7	2.7	4.0	2.7
Q_d	0.47	0.47	0.62	0.62	0.62	0.47	5.0	5.6	5.6	5.6
P_4	0.47	0.47	0.47	0.47	0.47	0.47	0.47	5.0	5.6	5.0
P_5	0.47	0.47	0.47	0.47	0.47	0.47	0.47	0.47	5.6	5.6
BP_4	0.47	0.47	0.47	0.47	0.47	0.47	0.47	0.57	0.47	5.0

The grey values are σ_{ij}, the rest values are ε_{ij}, for the green value, σ_{ij} is 0.47nm, for the pink value, ε_{ij} is 0.43nm.

The simulation box is 23.1nm*23.1nm*23.1nm and the number of total beads is N=108339. The temperature is kept at 300K and the pressure is maintained at 1 bar. The timestep is 20fs and the simulation run 600ns to get the equilibrium state. There is no need to use other methods to guarantee the electroneutrality of the system because the charges are already balanced (CTA^+ and Cl^-, Na^+ and Sal^-). The viscosity is calculated by changing the shape of the simulation box, which is a non-equilibrium molecular dynamics (NEMD) simulations of a continuously strained system.

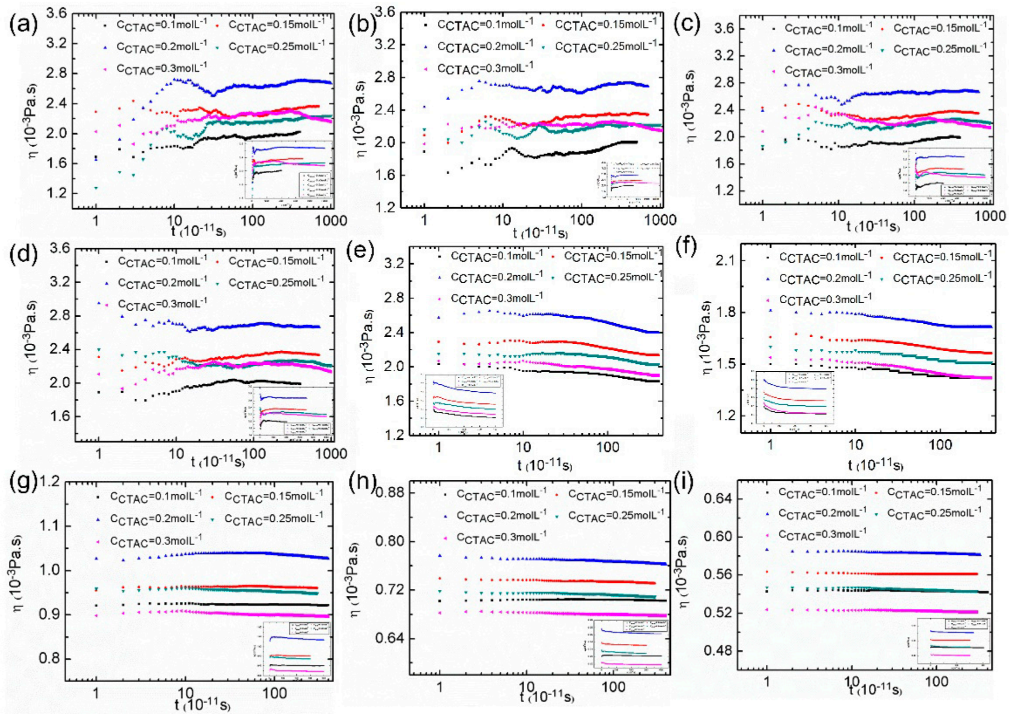

Figure S1. Shear viscosity versus time at various rates for PAM based solution. C_{PAM}=2.96× 10^{-3} mol·L^{-1}, Counter ion salt: NaSal, R=0.8, T=300K. Black square: C_{CTAC}=0.1 mol·L^{-1}. Red circle: C_{CTAC}=0.15 mol·L^{-1}. Blue triangle: C_{CTAC}=0.2 mol·L^{-1}. Dark cyan triangle: C_{CTAC}=0.25 mol·L^{-1}. Magenta triangle: C_{CTAC}=0.3 mol·L^{-1}. (a) shear rate is 7.79× 10^{8} s^{-1}, (b) shear rate is 8.65× 10^{8} s^{-1}, (c) shear rate is 1.08× 10^{9} s^{-1}, (d) shear rate is 1.3× 10^{9} s^{-1}. (e) shear rate is 4.32× 10^{9} s^{-1}, (f) shear rate is 1.3× 10^{10} s^{-1}, (g) shear rate is 4.32× 10^{10} s^{-1}, (h) shear rate is 8.65× 10^{10} s^{-1}, (i) shear rate is 1.73× 10^{11} s^{-1}.

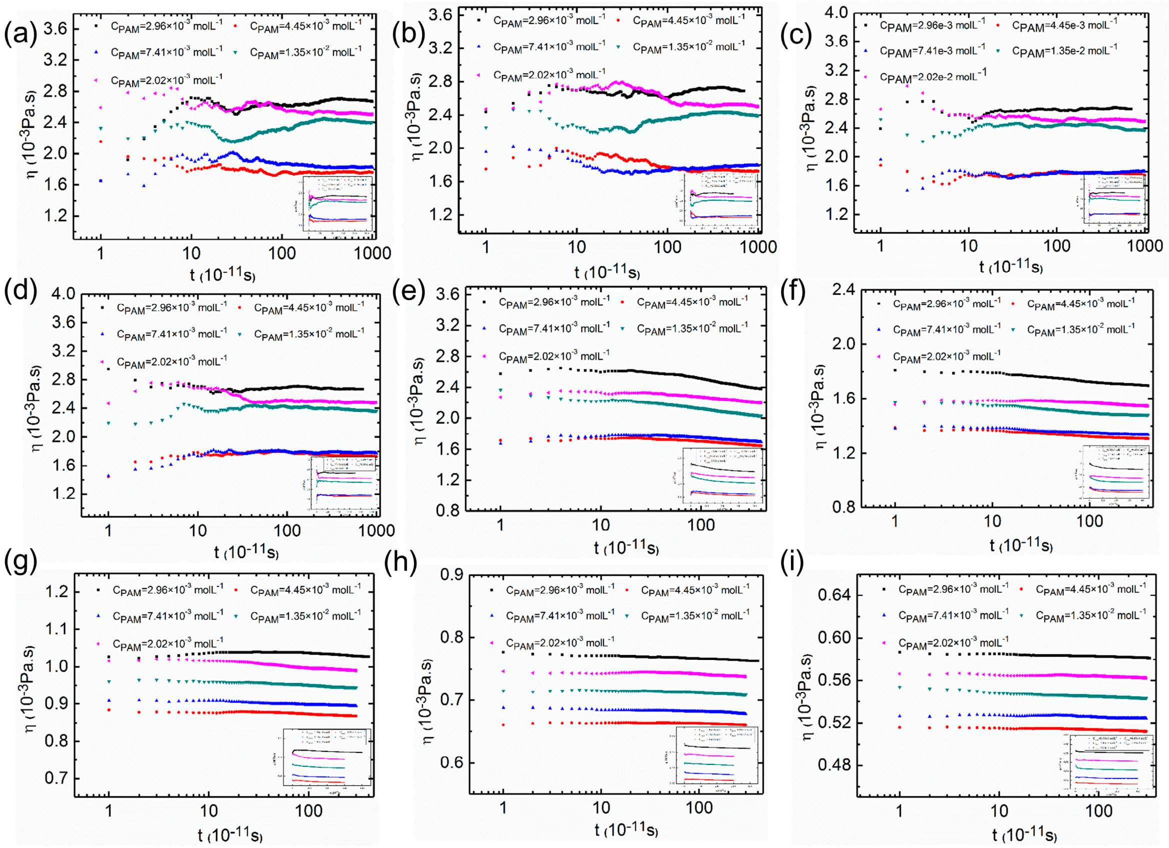

Figure S2. Shear viscosity versus time at low shear rates for CTAC based solution. C_{CTAC}=0.2 $mol\cdot L^{-1}$, counter ion salt: NaSal, R=0.8, T=300K. Black square: C_{PAM}=2.96× 10^{-3} $mol\cdot L^{-1}$. Red circle: C_{PAM}=4.45× 10^{-3} $mol\cdot L^{-1}$. Blue triangle: C_{PAM}=7.41× 10^{-3} $mol\cdot L^{-1}$. Dark cyan triangle: C_{PAM}=1.35× 10^{-2} $mol\cdot L^{-1}$. Magenta triangle: C_{PAM}=2.02× 10^{-2} $mol\cdot L^{-1}$. (a) shear rate is 7.79× 10^{8} s^{-1}, (b) shear rate is 8.65× 10^{8} s^{-1}, (c) shear rate is 1.08× 10^{9} s^{-1}, (d) shear rate is 1.3× 10^{9} s^{-1}. (e) shear rate is 4.32× 10^{9} s^{-1}, (f) shear rate is 1.3× 10^{10} s^{-1}, (g) shear rate is 4.32× 10^{10} s^{-1}, (h) shear rate is 8.65× 10^{11} s^{-1}, (i) shear rate is 1.73× 10^{11} s^{-1}.

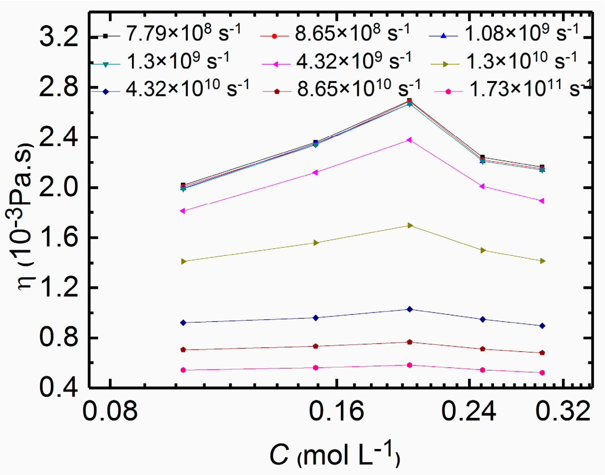

Figure S3. Shear viscosity *versus* the concentration of CTAC. The concentrations of CTAC are 0.1 $mol\cdot L^{-1}$, 0.15 $mol\cdot L^{-1}$, 0.2 $mol\cdot L^{-1}$, 0.25 $mol\cdot L^{-1}$, 0.3 $mol\cdot L^{-1}$, the concentration of PAM is 2.96× 10^{-3} $mol\cdot L^{-1}$. Counter ion salt is NaSal, R is 0.8, T=300K. Black square: shear rate is 7.79× 10^{8} s^{-1}; Red circle: shear rate is 8.65× 10^{8} s^{-1}; Blue triangle: shear rate is 1.08× 10^{9} s^{-1}; Dark cyan triangle: shear rate is 1.3× 10^{9} s^{-1}; Magenta triangle: shear rate is 4.32× 10^{9} s^{-1}; Dark yellow triangle: shear rate is 1.3× 10^{10} s^{-1}; Navy rhombus: shear rate is 4.32× 10^{10} s^{-1}; Wine pentagon: shear rate is 8.65× 10^{10} s^{-1}; Pink hexagon: shear rate is 1.73× 10^{11} s^{-1}.

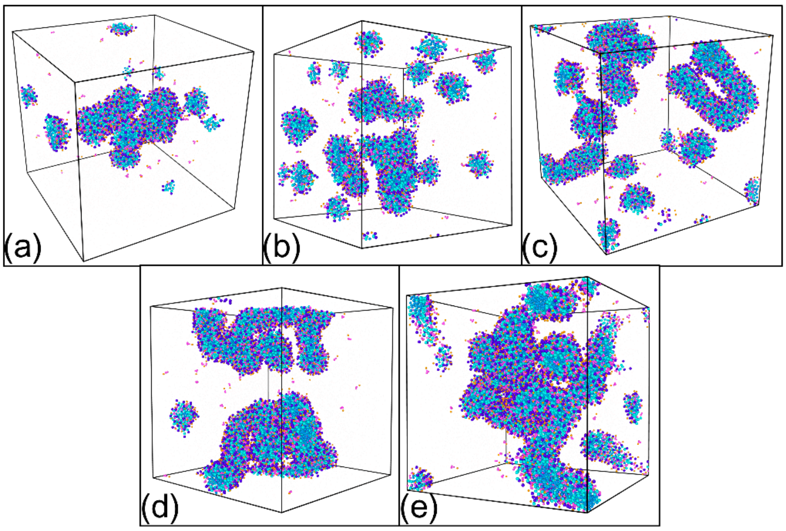

Figure S4. The inner micelle structures in stable PAM based solutions (initial equilibrium state). (a) C_{CTAC}=0.1 $mol\cdot L^{-1}$, (b) C_{CTAC}=0.15 $mol\cdot L^{-1}$, (c) C_{CTAC}=0.2 $mol\cdot L^{-1}$, (d) C_{CTAC}=0.25 $mol\cdot L^{-1}$, (e) C_{CTAC}=0.3 $mol\cdot L^{-1}$. C_{PAM}=2.96× 10^{-3} $mol\cdot L^{-1}$, counter ion salt is NaSal, R is 0.8, T=300K. Color scheme: purple, hydrophilic part of CTAC; cyan and blue, hydrophobic tail of CTAC; magenta, aromatic ring of Sal^{-}; khaki, charge group of Sal^{-}.PAM acidammide group (red), PAM carbon backbone (green), Na^{+} (yellow), Cl^{-} (dark green), Water sites (reddish) and antifreeze water sites (grey) were hidden for clear display.

From the Figure S3 and S4, we can find that when the concentration of CTAC is low, CTAC will form small size micelles such as spherical micelles and small wormlike micelles, (Figure S4(a)and (b)). With the increase of CTAC concentration, CTAC will form large size of micelles, such as wormlike micelles and branched micelles.

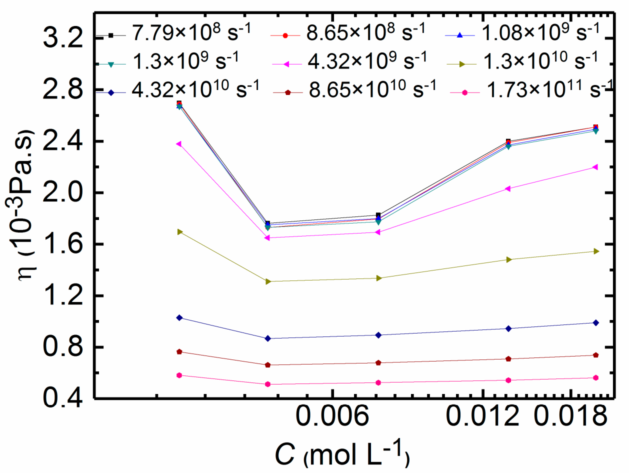

Figure S5. Shear viscosity *versus* the concentration of PAM. The concentrations of PAM are 2.96× 10^{-3} mol·L^{-1}, 4.45× 10^{-3} mol·L^{-1}, 7.41× 10^{-3} mol·L^{-1}, 1.35× 10^{-2} mol·L^{-1}, 2.02× 10^{-2} mol·L^{-1}, the concentration of CTAC is 0.2 mol·L^{-1}. Counter ion salt is NaSal, R is 0.8, T=300K. Black square: shear rate is 7.79× 10^{8} s^{-1}; Red circle: shear rate is 8.65× 10^{8} s^{-1}; Blue triangle: shear rate is 1.08× 10^{9} s^{-1}; Dark cyan triangle: shear rate is 1.3× 10^{9} s^{-1}; Magenta triangle: shear rate is 4.32× 10^{9} s^{-1}; Dark yellow triangle: shear rate is 1.3× 10^{10} s^{-1}; Navy rhombus: shear rate is 4.32× 10^{10} s^{-1}; Wine pentagon: shear rate is 8.65× 10^{10} s^{-1}; Pink hexagon: shear rate is 1.73× 10^{11} s^{-1}.

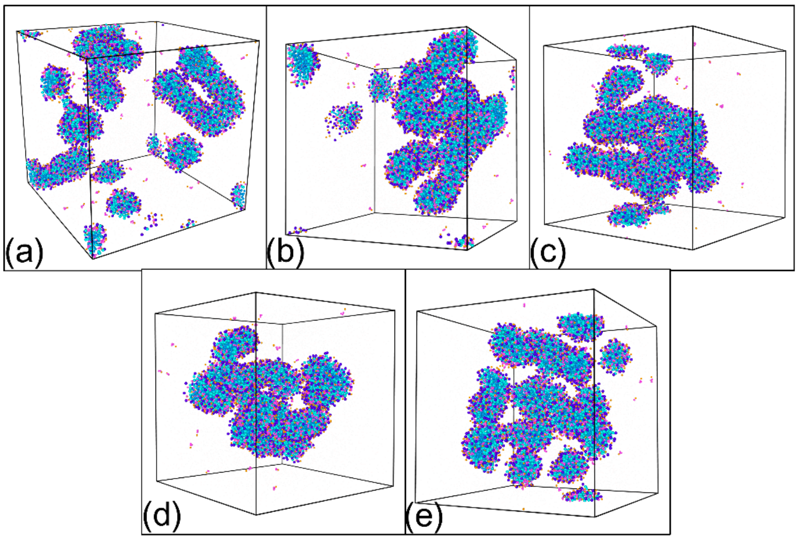

Figure S6. The inner micelle structures in CTAC based solutions (initial equilibrium state). (a) C_{PAM}=2.96× 10^{-3} mol·L^{-1}, (b) C_{PAM}=4.45× 10^{-3} mol·L^{-1}, (c) C_{PAM}=7.41× 10^{-3} mol·L^{-1}, (d) 1.35× 10^{-2} mol·L^{-1}, (e) 2.02× 10^{-2} mol·L^{-1}. C_{CTAC}=0.2 mol·L^{-1}, counter ion salt is NaSal, R is 0.8, T=300K. Color scheme: purple, hydrophilic part of CTAC; cyan and blue, hydrophobic tail of CTAC; magenta, aromatic ring of Sal^{-}; khaki, charge group of Sal^{-}.PAM acidammide group (red), PAM carbon backbone (green), Na^{+} (yellow), Cl^{-} (dark green), Water sites (reddish) and antifreeze water sites (grey) were hidden for clear display.

From the Figure S5 and S6, we can see that with the increase of PAM concentration, the micelle size first has an evident increase, small spherical micelles disappear and large branched micelles appear. Then with the further increase of PAM concentration, the micelle size decrease and small micelles appear.

The Table of Contents entry

The chemical-physical interactions within multi-phase PAM/CTAC/NaSal mixtures were explored with a NEMD based numerical approach. The "two peaks" phenomenon was discovered first time in simulation after analysing the time dependent shear viscosity and the corresponding inner structural evolution between different phases under different shear rates in PAM based solutions.

Keyword: Rheology, shear viscosity, shear rate, molecular dynamic

Dongjie Liu, Yong Li, Fei Liu, Wenjing Zhou, Xiaoteng Liu, Fei Chen, Ben Bin Xu*, Jinjia Wei**

Interfacial Interaction Enhanced Rheological Behavior in PAM/CTAC/Salt Aqueous Solution - A Coarse-Grained Molecular Dynamics Study

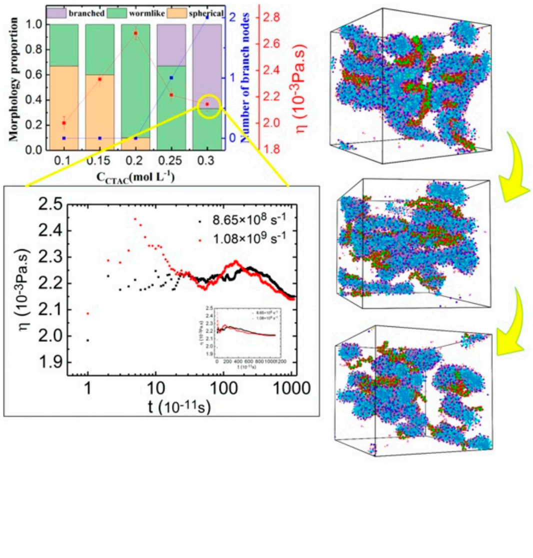

Supplement: Supplementary file 1 [file polymers-12-00265-s001.pdf]
